# Supplementary material for: Whole Transcription Profile of Responders to Anti-TNF Drugs in Pediatric Inflammatory Bowel Disease
Source: Pharmaceutics. 2021 Jan 8;13(1):77. doi: 10.3390/pharmaceutics13010077 (PMC7830359; doi:10.3390/pharmaceutics13010077)
Supplement: Supplementary file 1 [file pharmaceutics-13-00077-s001.pdf]

pharmaceutics

Supplement

Whole Transcription Profile of Responders to Anti-TNF Drugs in Pediatric Inflammatory Bowel Disease

**Sara Salvador-Martín [1,†], Bartosz Kaczmarczyk [1,†], Rebeca Álvarez [2], Víctor Manuel Navas-López [3], Carmen Gallego-Fernández [4], Ana Moreno-Álvarez [5], Alfonso Solar-Boga [5], Cesar Sánchez [6], Mar Tolin [6],
Marta Velasco [7], Rosana Muñoz-Codoceo [7], Alejandro Rodriguez-Martinez [8], Concepción A. Vayo [9],
Ferrán Bossacoma [10], Gemma Pujol-Muncunill [11], María J. Fobelo [12], Antonio Millán-Jiménez [13], Lorena Magallares [14], Eva Martínez-Ojinaga [14], Inés Loverdos [15], Francisco J. Eizaguirre [16], José A. Blanca-García [17],
Susana Clemente [18], Ruth García-Romero [19], Vicente Merino-Bohórquez [20], Rafael González de Caldas [21],
Enrique Vázquez [2], Ana Dopazo [2], María Sanjurjo-Sáez [1] and Luis A. López-Fernández [1,*]**

Table S1. Characteristics of patients selected for RNAseq. .

Characteristic	Overall (*n* = 12)	Responders (*n* = 6)	Non-Responders (*n* = 6)	*p* Value
**Gender**				
Male, *n* (%)	8 (66.7%)	4 (66.7%)	4 (66.7%)	1
Female, *n* (%)	4 (33.3%)	2 (33.3%)	2 (33.3%)	
**Age (years)**				
At diagnosis, median (IQR, range)	10 (3.53, 0.7-13)	9.85 (2.38, 7.9-12)	10.2 (7.2, 0.7-13)	0.630
At start of treatment, median (IQR, range)	10.2 (3.1, 1.1-14.1)	9.9 (1.83, 7.9-12.8)	11.4 (6.25, 1.1-14.1)	0.956
**Type of IBD**				
CD,*n* (%)	9 (75%)	4 (66.7%)	5 (83.3%)	1
UC,*n* (%)	3 (25%)	2 (33.3%)	1 (16.7%)	
**Type of Anti-TNF**				
Infliximab,*n* (%)	8 (66.7%)	4 (66.7%)	4 (66.7%)	1
Adalimumab,*n* (%)	4 (33.3%)	2 (33.3%)	2 (33.3%)	
**PCDAI at start of treatment, median (IQR, range)**	27.5 (20, 7.5-52.5)	35 (21.25, 27.5-52.5)	15 (12.5, 7.5-30)	0.025**
**PUCAI at start of treatment, median (IQR, range)**	45 (40-60)*	50 (40-60)*	45*	-

**CRP at start of treatment, median (IQR, range)**	22.3 (25.1, 0.7-52.3)	28.8 (30.38, 0.7-52.3)	10.58 (19.15, 6.12-27.5)	0.121
**FC at start of treatment, median (IQR, range)**	1800 (2392, 225.1-3956)	2700 (1934.73, 225.1-3956)	1319 (1352, 288-1800)	0.075
**Concomitant immunomodulator at start of treatment**				
Azathioprine,*n* (%)	7 (58.3%)	4 (66.7%)	3 (50%)	
Methotrexate,*n* (%)	2 (16.7%)	2 (33.3%)	0	0.076
None,*n* (%)	3 (25%)	0	3 (50%)	

IBD, inflammatory bowel disease; CD, Crohn's disease; UC, ulcerative colitis; IQR, interquartile range; PCDAI, Pediatric Crohn's Disease Activity Index; PUCAI, Pediatric Ulcerative Colitis Activity Index; CRP, C-reactive protein; FC, fecal calprotectin. * IQR not applicable. ** p value < 0.05.

Table S2. Top diseases and biofunctions of differentially expressed between responders and non-responders prior anti-TNF treatment initiation.

Cathegory	Process	p value range
Diseases and disorders	Developmental disorder	$1.96 \times 10^{-2} – 9.23 \times 10^{-15}$
	Hereditary disorder	$1.48 \times 10^{-2} – 9.23 \times 10^{-15}$
	Metabolic disease	$1.25 \times 10^{-2} – 9.23 \times 10^{-15}$
	Neurological disease	$1.96 \times 10^{-2} – 9.23 \times 10^{-15}$
	Ophthalmic disease	$5.27 \times 10^{-2} – 9.23 \times 10^{-15}$
Molecular and Cellular Function	Cell-to-cell signaling interaction	$1.96 \times 10^{-2} – 4.30 \times 10^{-6}$
	Cellular movement	$1.96 \times 10^{-2} – 4.22 \times 10^{-6}$
	Cell signaling	$1.93 \times 10^{-2} – 1.22 \times 10^{-6}$
	Cellular assembly and organization	$1.70 \times 10^{-2} – 5.04 \times 10^{-6}$
	Cellular growth and proliferation	$1.96 \times 10^{-2} – 5.41 \times 10^{-6}$
Physiological System development and function	Hematological system development and function	$1.96 \times 10^{-2} – 4.30 \times 10^{-9}$
	Immune cell trafficking	$1.96 \times 10^{-2} – 4.30 \times 10^{-9}$
	Lymphoid tissue structure and development	$1.93 \times 10^{-2} – 1.10 \times 10^{-5}$
	Cell-mediated immune response	$1.83 \times 10^{-2} – 2.52 \times 10^{-5}$
	Organismal functions	$6.58 \times 10^{-2} – 7.43 \times 10^{-5}$

Table S3. Top diseases and biofunctions of differentially expressed between responders and non-responders after two weeks of anti-TNF treatment initiation.

Category	Process	*p* value range
Diseases and disorders	Inflammatory response	$1.30 \times 10^{-2} - 5.74 \times 10^{-23}$
	Organismal injury and abnormalities	$1.30 \times 10^{-2} - 5.21 \times 10^{-6}$
	Dermatological diseases and conditions	$8.55 \times 10^{-3} - 5.86 \times 10^{-6}$
	Connective tissue disorders	$1.30 \times 10^{-2} - 1.40 \times 10^{-5}$
	Inflammatory disease	$1.30 \times 10^{-2} - 1.40 \times 10^{-5}$
Molecular and Cellular Function	Cellular function and maintenance	$1.19 \times 10^{-2} - 5.11 \times 10^{-19}$
	Cellular movement	$1.19 \times 10^{-2} - 1.13 \times 10^{-7}$
	Cellular assembly and organization	$1.02 \times 10^{-2} - 5.99 \times 10^{-5}$
	Cell-to-death signaling and interaction	$1.30 \times 10^{-2} - 7.98 \times 10^{-5}$
	Cell death and survival	$1.19 \times 10^{-2} - 1.02 \times 10^{-4}$
Physiological System development and function	Humoral immune response	$1.23 \times 10^{-2} - 5.74 \times 10^{-23}$
	Immune cell trafficking	$1.20 \times 10^{-2} - 1.13 \times 10^{-7}$
	Connective tissue development and function	$1.02 \times 10^{-2} - 7.98 \times 10^{-5}$
	Tissue morphology	$1.02 \times 10^{-2} - 3.01 \times 10^{-4}$
	Endocrine system development and function	$1.19 \times 10^{-2} - 4.31 \times 10^{-4}$
